# Relationship between Coping with Interpersonal Stressors and Depressive Symptoms in the United States, Australia, and China: A Focus on Reassessing Coping

**DOI:** 10.1371/journal.pone.0109644

**Published:** 2014-10-09

**Authors:** Tsukasa Kato

**Affiliations:** Toyo University, Department of Social Psychology, Bunkyo-ku, Tokyo, Japan; Radboud University, Netherlands

## Abstract

**Objective:**

Reassessing coping involves efforts to wait patiently for an appropriate opportunity to act or for a change or improvement in the situation, and can be observed in individuals encountering a stressful relationship event. It was hypothesized that reassessing coping would be negatively associated with depressive symptoms.

**Methods:**

A cross-sectional Web-based survey was conducted in order to test this hypothesis by examining relationships between coping strategies including reassessing coping, distancing coping and constructive coping for stressful relationship events and depressive symptoms. Participants were 1,500 individuals recruited from the general populations of the United States, Australia, and China.

**Results:**

Structural equation modeling analysis revealed that scores on coping strategies predicted depressive symptom scores in the samples from all three countries with medium or large effect sizes. Further, the beta values for reassessing coping scores were negative and significant in all samples, indicating that the hypothesis was supported for each of the population samples surveyed. In addition, distancing coping, which reflects strategies that attempt to actively damage, disrupt, and dissolve a stressful relationship, was associated with high levels of depressive symptoms.

**Conclusions:**

Reassessing coping for interpersonal stressors was be negatively associated with depressive symptoms in sample from general populations of the United States, Australia, and China.

## Introduction

Several researchers [1]–[4] have proposed mechanisms by which interpersonal stressors increase the risk of depression and provided evidence for their relationships. The World Health Organization [5] states that certain mental and behavioral disorders, such as depression and anxiety, can result from failing to cope adaptively with a stressor. According to the transactional theory proposed by Lazarus et al. [6], [7], coping is defined as “constantly changing cognitive and behavioral efforts to manage specific external and/or internal demands that are appraised as taxing or exceeding the resources of the person” (p. 141). This theory hypothesizes that coping behavior affects psychological functioning, including depressive symptoms, and has been supported by numerous studies [6].

Kato [8] defined interpersonal stressors as “stressful episodes between two or more people that involve quarrels, arguments, negative attitudes or behavior, an uncomfortable atmosphere during a conversation or activity, and concern about hurting others’ feelings” (p. 100); he proposed three types of coping strategies, based on the transactional theory, for dealing with interpersonal stressors. According to research on how college students and workers deal with interpersonal stressors, the following coping strategies were identified: reassessing coping, distancing coping, and constructive coping. Although coping strategies individuals employ and the effects on psychological distress are dependent on the nature of the stressors [6], [7], conventional research has measured and categorized coping strategies for interpersonal stressors using a broadly applicable coping scale [8], such as the Ways of Coping Questionnaire (WCQ) [9]. Therefore, Kato’s approach [8] presented unique coping strategies for interpersonal stressors. Thus, focusing on these three coping strategies proposed by Kato [8], in particular the strategy of reassessing coping, we examined the relationships between coping strategies for interpersonal stressors and depressive symptoms in general populations of the United States, Australia, and China.

### Reassessing coping

Reassessing coping refers to efforts to wait patiently for an appropriate opportunity to act or for a change or improvement in the situation [8]. This type of coping is an active strategy that involves exercising self-control, avoiding premature action, and waiting until an appropriate opportunity arises. Therefore, reassessing coping is distinct from avoidant coping, which involves avoiding stressful problems or situations. Previous studies have found a non-significant negative correlation between reassessing coping and avoidant coping [8]. Kato [10] stated that reassessing coping enables people to take time to deal with stressful relationships, attain a better grasp of the situation, control their emotions, and consider appropriate countermeasures. Additionally, reassessing coping can influence other individuals or parties involved within the stressful relationship, helping to change their mental state, and thus permitting them to adopt a calmer, more accepting attitude toward the stressful relationship as well. In short, reassessing coping increases the likelihood that the situation will improve.

Thus, some studies have suggested that reassessing coping attenuates psychological distress in response to stressful relationships. For example, in a sample of college students, reassessing coping was negatively associated with depressive symptoms, anxiety, and general distress [8]. Correlations between reassessing coping and good psychological functioning, including experiencing fewer depressive symptoms, have been reported by other studies conducted among full-time workers in dealing with workplace interpersonal stress [10] and among hospital nursing staff in relationships with patients as stressors [11]. Moreover, a previous study [8] showed that reassessing coping incrementally predicted depressive symptoms beyond the effects of other popular coping strategies (i.e., 8 strategies of the WCQ) and conflict management styles (i.e., 5 styles categorized by the dual-concern model [12], [13]) on depressive symptoms.

Although the aforementioned findings on the effects of reassessing coping on depressive symptoms were obtained through research performed specifically in Japan, several studies suggest that these findings may be supported for Western and other populations. For example, clinical interventions based on mindfulness [14]–[16], detached mindfulness [17], or meta-cognitive awareness [18], propose these strategies as primary skill that individuals should develop. These skills, which allow distancing oneself from negative emotions when they are evoked [16], have been shown to reduce psychological distress and promote well-being [for reviews, 17,19]. Thus, it is likely that distancing oneself from stressful situations represents a coping strategy that could reduce depressive symptoms. Therefore, we hypothesized that reassessing coping would be associated with a lower level of depressive symptoms in populations of other countries as well.

In the current study, we focused on reassessing coping among three coping strategies proposed by Kato [8], because no other coping style has proven to be effective in reducing psychological dysfunction including depressive symptoms in interpersonal stress. For example, Penley et al.’s [20] meta-analytic review of 11 coping types found that all coping styles were either negatively or non-significantly related to physical or psychological health outcomes with respect to interpersonal relationships as stressors.

### Distancing coping

Distancing coping reflects strategies that attempt to actively damage, disrupt, and dissolve a stressful relationship (e.g. avoiding contact with the person and ignoring the person); that is, distancing in this coping style means intentionally breaking off relations with another individual involved within the stressful relationship. Therefore, this coping strategies differs from the concept of distancing used in mindfulness-based clinical interventions.

Distancing coping may lead to poor interpersonal relationships [8], and the deterioration of these relationships can produce psychological and physiological dysfunctions [for a review, 21]. Distancing coping was indeed found to be positively correlated with depressive symptoms, anxiety, burnout, insomnia, and general psychological distress in a study involving Japanese students [8], workers [10], [11], and hospital nursing staffs [11]. Therefore, we hypothesized that distancing coping would be associated with a higher level of depressive symptoms in populations of other countries as well.

### Constructive coping

Another strategy, constructive coping, involves efforts that actively seek to improve, maintain or sustain a relationship without aggravating others (e.g. reflecting on one’s own conduct and trying to understand the other person’s feelings) and emphasizes respecting and living in harmony with others. Previous studies in Japanese samples have found positive but no-significant correlations between constructive coping and depressive symptoms [8], [10]. Likewise, some studies [22], [23] have indicated no significant correlation between coping strategies similar to constructive coping and depressive symptoms but positive. Other studies on coping strategies similar to constructive coping [24] have found that such coping is significantly associated with increased psychological distress. Therefore, we hypothesized that there would be positive or no-significant correlation between constructive and depressive symptoms.

### Cross-cultural differences in coping strategies

In the present study, we also examined cross-cultural differences in the frequency with which reassessing coping is used, as individuals from different cultures are known to adopt different coping strategies [for a review, 25]. For instance, Triandis [26] suggested that the following characteristics are distinctive of collectivistic cultures: over cautiousness, hypervigilance, avoidance of decisions and interpersonal conflicts, and increased likelihood of using silence, ambiguity, and indirect messages during communication― all of which, according to Kato [8], are included in reassessing coping. Constructive coping may also include characteristics of collectivistic cultures as they emphasize respecting and living in harmony with others, and how one behaves is often based on what one perceives to be the thoughts, feelings, and actions of others [26]. Oyserman et al.’s meta-analysis [27] in various societies revealed that collectivism was lower in Australia, but higher in China, as compared to the United States. Therefore, we hypothesized that reassessing coping and constructive coping would be most frequently used in China, followed by the United States, and Australia.

We selected the United States and Australian samples as representing from individualistic cultures and the Chinese sample as a collectivistic cultures for a Web-based survey. The United States and China are the most highly populated countries among individualistic and collectivistic cultures, respectively. Australia has been dominated by immigration that reflects a multicultural mix representing the globe, and more than a quarter of the Australian population was born in other countries.

### Gender differences in coping strategies

Gender differences in coping have not yet been established conclusively; however, women use more coping strategies in general than men [for a review, 28]. Women also encounter higher levels of stressful events related to interpersonal relationships [for reviews, 29,30] and are more likely to respond more sensitively [for a review, 4,30]. Therefore, women may attempt to use more coping strategies in general also for interpersonal stressors than men. As a result, we hypothesized that women would employ distracting coping and constructive coping more frequency than men. As reassessing coping is a waiting strategy without approach to another individual involved within the stressful relationships, we hypothesized that gender differences in this particular form of coping would be absent in the current study. In fact, no significant gender difference in reassessing coping was found in Japanese samples [8].

## Methods

### Participants

#### United States sample

This sample comprised 246 men and 254 women aged 18 to 79 years (mean age 45.4, *SD* = 15.3). Approximately 78.6% were Caucasian, 7.2% were African American, 5.6% were Asian American, 4.8% were Hispanic, and 3.6% were of other ethnicities; one person preferred not to say. Further, about 51.4% of the participants were married, 31.2% had never been married, and 17.4% were divorced, separated, or widowed.

#### Australian sample

The Australian sample included 239 men and 261 women aged 18 to 79 years (mean age 45.4, *SD* = 14.8). Approximately 55.8% were Caucasian, 29.2% were European Australian, 7.8% were Asian Australian, and 6.6% were of other ethnicities; six persons preferred not to say. In addition, approximately 54.2% were married and 32.2% had never been married, while 13.6% were divorced, separated, or widowed.

#### Chinese sample

Individuals in this sample included 263 men and 237 women aged 19 to 76 years (mean age 40.1, *SD* = 11.9). Approximately 98.0% were Chinese, and 2% were of other ethnicities. In terms of marital status, 87.0% were married, 11.6% had never been married, and 1.4% were divorced, separated, or widowed.

### Procedure

All participants were recruited through a Web-based survey. Data were collected by the polling organization Rakuten Research (Tokyo, Japan), through their Web panel (see http://research.rakuten.co.jp/en/) of over 4.01, 0.83, and 1.43 million members in the United States, Australia, and China, respectively, who had registered and received one ID per person.

The details of the survey were sent to potential participants, who ranged in age from 18 to 79 years, through an e-mail in early December 2013. If potential participants agreed to participate in this survey, they clicked on another link to view the survey, which began once they entered their ID. Participants could not skip any questionnaire items. The data were collected so that a sample was almost evenly divided by gender and age in each country. A chi-square test showed that no significant differences in gender between the three samples were found at *p* <.05. A Kruskal-Wallis rank analysis revealed that the median age in the Chinese sample was significantly lower than in the United States and Australian samples, but no significant difference between was United States and Australian was found at *p* <.05. The project, including the Web-based survey, was approved by the Institutional Ethics Committee of the Department of Social Psychology at Toyo University in Japan.

### Measures

#### Coping with interpersonal stressors

The Interpersonal Stress Coping Scale (ISCS) [8], which was designed to measure coping strategies for interpersonal stressors, was used. The ISCS consists of three 5-item subscales evaluating reassessing coping (e.g., taking a pragmatic view of the matter, deciding not to take the matter too seriously), distancing coping (e.g., avoiding contact with the person, ignoring the person), and constructive coping (e.g., reflecting on one’s own conduct, trying to understand the other person’s feelings). A previous study [8] reported that confirmatory factor analyses (CFAs) showed good fit to the three-factor model for the ISCS, but poor fit to other models (e.g., one-factor model and some two factor models). The score of each of the three strategies is associated with the theoretically related constructs [8]. The validity for the ISCS score was previously established only in Japanese samples. Cronbach’s alphas for scores on reassessing, distancing, and constructive coping were.805 (95% CI [.780,.828]),.839 (95% CI [.823,.854]), and.733 (95% CI [.699,.762]), respectively [8]; the number of independent alphas was eight (*N* = 3,686).

The instructions were as follows: “Please recall the specifics of your own experiences of stress due to interpersonal relationships. These may include quarreling with others, being talked about behind your back, feeling awkward while speaking, and worrying if you have hurt someone’s feelings. Please read each item and indicate to what extent you used that strategy in the situations you encountered.” Participants rated the extent to which they used each item on a 4-point Likert scale ranging from 0 (*did not use*) to 3 (*used a great deal*). In the present study, trait coping strategies were measured.

#### Depressive symptoms

The Center for Epidemiologic Studies’ Depression Scale (CES-D) [31], a 20-item self-report scale, was used to measure depressive symptoms in this study. Development of the CES-D was originally based on the American population [31]; however, the validity and reliability of CES-D scores have also been established in a number of other populations, including those of Australia [32] and China [33]. Participants rated each item according to their experiences within the past week on a 4-point Likert scale ranging from 1 (*rarely or none of the time*) to 4 (*most or all of the time*).

### Data analysis

First, three measurement invariances of the ISCS (3 factors, 5 items per factor) across the three samples were tested using a mean and covariance structure (MACS) analysis, based on CFA models, in order to compare mean ISCS subscale scores: configural invariance (equivalence of factor structure across groups), metric invariance (equivalence of factor loadings across groups), and invariance in uniqueness (equivalence of error variances across groups). Configural invariance served as a baseline model. Scalar invariance (equivalence of intercepts across groups) was not tested because it was unclear as to whether observed mean differences reflected lack of measurement invariance or simple actual differences [34].

Based on guidelines proposed by Hu and Bentler [35], root-mean-square error of approximation (RMSEA) values of.06 or lower, standardized root-mean-square residual (SRMR) values of.08 or lower, and comparative fit index (CFI) values of.95 or greater were considered good fits; CFI values greater than.90 were deemed acceptable [35]. A change in CFI (ΔCFI) value equal to or less than.01 served as a practical indicator of substantial improvement in fit [36]. Although chi-square statistics (i.e., χ^2^ and Δχ^2^) are known to be sensitive to sample size, we provided these statistics as they have traditionally been used as indicators of goodness-of-fit for CFA.

Second, our hypothesis on the relationship between three coping strategies and depressive symptoms was tested using a multiple group analysis, which enabled cross-cultural and gender comparisons of the relationship. Structural equation modeling (SEM) was conducted using the Amos 22 software [37].

## Results

### Measurement invariances of the ISCS

The MACS was conducted to test the measurement invariances of the ISCS. Goodness-of-fit statistics pertaining to the three models are shown in Table 1. The RMSEA and SRMR values for the three models were less than the cutoff criteria of.06 and.08, respectively; the CFI values were greater than the cutoff criterion of.90, which indicated acceptable fit. ΔCFI values of −.004 and −.004 were both less than the cutoff criterion of.01 [36]. These results suggested that the three-factor model for the ISCS had the same factor structure, with similar factor loadings and error variances across all three samples.

**Table 1:** Goodness-of-fit Indexes for the three-factor model for the ISCS.

Model	χ^2^	*df*		CFI	RMSEA	SRMR	Δχ^2^	Δ*df*	ΔCFI
Configural invariance	849.36	261	***	.915	.039	.052			
Metric invariance	905.74	285	***	.911	.038	.053	56.38	24	.004
Invariance in uniqueness	1154.62	327	***	.911	.041	.059	305.26	66	.004

*** *p*<.001.

### Cross-cultural differences in coping with interpersonal stress

Means, standard deviations, and alpha coefficients for all variables are shown in Table 2. To test cross-cultural differences with regard to each coping strategy, 2 (gender) × 3 (countries) analyses of variance (ANOVAs) were conducted, with the ISCS scores for each coping strategy as the dependent variable. The ANOVA on reassessing coping revealed that only the main effect of country was significant (*F*(2, 1494) = 48.35, *p*<.001, η_p_^2^  = .061); however, the interaction between gender and country (*F*(2, 1494) = 2.92, *p* = .054, η_p_^2^  = .004) as well as the main effect of gender (*F*(1, 1494) = 0.06, *p* = .81, η_p_^2^  = .001) were not significant at *p*<.05. Furthermore, Bonferroni post-hoc tests showed that reassessing coping scores were significantly higher in China (*M* = 8.66, *SD* = 2.65) than in the United States (*M* = 7.57, *SD* = 3.21) and Australia (*M* = 6.81, *SD* = 3.16), in that order.

**Table 2:** Means, standard deviations, and alphas for scores of each coping strategy and depressive symptoms.

			United States				Australia				China		
Variable													
		n	Mean	SD	α	n	Mean	SD	α	n	Mean	SD	α
Reassessing coping	Men	246	7.86	3.03	.66	239	6.64	3.32	.75	263	8.60	2.56	.65
	Women	254	7.30	3.35	.73	261	6.96	3.00	.70	237	8.73	2.75	.70
Distancing coping	Men	246	5.70	3.71	.83	239	5.22	3.74	.85	263	5.30	2.81	.75
	Women	254	6.11	4.19	.88	261	5.52	3.89	.86	237	5.70	2.70	.71
Constructive coping	Men	246	6.93	3.35	.79	239	5.80	3.19	.78	263	7.83	2.83	.76
	Women	254	7.51	3.71	.83	261	6.67	3.19	.77	237	8.30	3.07	.79
Depressive symptoms	Men	246	36.22	11.16	.91	239	34.65	11.21	.92	263	35.32	9.26	.88
	Women	254	38.72	13.51	.93	261	36.70	11.98	.93	237	35.85	9.81	.90

In the ANOVA on distancing coping, significant main effects of gender (*F*(1, 1494) = 4.12, *p*<.05, η_p_^2^  = .003) and country (*F*(2, 1494) = 3.06, *p*<.05, η_p_^2^  = .004), but no significant interaction (*F*(2, 1494) = 0.04, *p* = .096, η_p_^2^  = .001) were observed. Bonferroni post-hoc tests revealed that distancing coping scores for women (*M* = 5.78, *SD* = 3.68) were significantly higher than those for men (*M* = 5.40, *SD* = 3.43), whereas differences between the three countries were not significant.

The ANOVA on constructive coping also revealed significant main effects of gender (*F*(1, 1494) = 14.70, *p*<.001, η_p_^2^  = .010) and country (*F*(2, 1494) = 39.82, *p*<.001, η_p_^2^  = .051) but no significant interaction (*F*(2, 1494) = 0.52, *p* = .595, η_p_^2^  = .001). Through Bonferroni post-hoc tests, constructive coping scores were found to be significantly higher in China (*M* = 8.05, *SD* = 2.95) than in the United States (*M* = 7.23, *SD* = 3.55) and Australia (*M* = 6.26, *SD* = 3.22), in that order. Additionally, women (*M* = 7.47, *SD* = 3.40) scored significantly higher than men (*M* = 6.89, *SD* = 3.23) on this type of coping.

### Coping strategies and depressive symptoms

**Figure 1:**
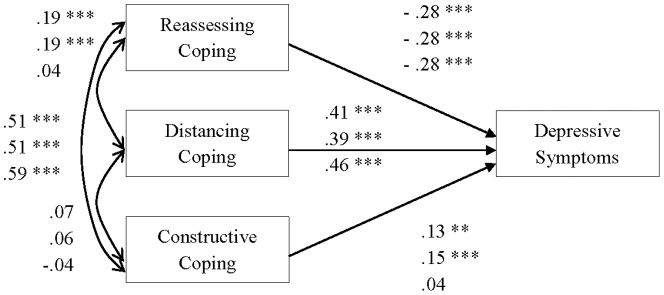
Path coefficients (β: Standardized partial regression coefficients) in structural equation modeling. The upper, center, and lower values are the beta weights in the United States, Australian, and Chinese samples, respectively. Three asterisks (***) and two asterisks (**) are p<.001 and p<.01, respectively.

To test our hypothesis on the relationship between reassessing coping and depressive symptoms, a multiple group analysis, which enabled cross-cultural comparisons of the relationship, was conducted using maximum likelihood estimation (see Figure 1). For all samples, the *R* ^2^ values were significant at the *p*<.001 level (United States: *R* ^2^  = .19, *F* (3,496) = 39.50, Cohen’s *f* ^2^  = .24; Australia: *R* ^2^  = .18, *F* (3,496) = 35.41, Cohen’s *f* ^2^  = .21; China: *R* ^2^  = .26, *F* (3,496) = 58.80, Cohen’s *f* ^2^  = .36), indicating that significant proportions of the variance in depressive symptom scores were accounted for by scores of each coping strategy (see Table 3). The beta weights for reassessing coping scores in all samples were significant and negative in the SEM analysis with depressive symptom scores (United States: *β* = −.28, *p*<.001; Australia: *β* = −.28, *p*<.001; China: *β* = −.28, *p*<.001). There were no significant differences in coping scores between all samples at the *p*<.05 level (*z* = 0.32, 0.43, and 0.11; *p* = .37,.33, and.46; United States vs. Australia, United States vs. China, and Australia vs. China).

**Table 3:** Structural equation modeling analyses predicting depressive symptoms scores from scores of each coping strategy.

Predictor							95% CL	
	R^2^	B	SE B	β	t		LL	UL
United States	.19	***	*N* = 500					
Reassessing coping		− 1.10	0.18	−.28	−5.94	***	−1.46	−0.73
Distancing coping		1.29	0.13	.41	10.05	***	1.04	1.55
Constructive coping		0.46	0.16	.13	2.77	**	0.13	0.78
Australia	.18	***	*N* = 500					
Reassessing coping		−1.01	0.18	−.28	−5.73	***	−1.36	−0.67
Distancing coping		1.19	0.13	.39	9.46	***	0.95	1.44
Constructive coping		0.56	0.17	.15	3.28	***	0.22	0.90
China	.26	***	*N* = 500					
Reassessing coping		−0.99	0.17	−.28	−5.74	***	−1.33	−0.65
Distancing coping		1.57	0.13	.46	11.84	***	1.31	1.83
Constructive coping		0.12	0.15	.04	0.78		−0.18	0.43
Women	.17	***	*N* = 752					
Reassessing coping		−0.87	0.16	−.23	−5.57	***	−1.17	−0.56
Distancing coping		1.26	0.11	.39	11.62	***	1.05	1.48
Constructive coping		0.25	0.14	.07	1.77		−0.03	0.53
Men	.24	***	*N* = 748					
Reassessing coping		−1.20	0.13	−.35	−8.98	***	−1.46	−0.93
Distancing coping		1.39	0.10	.45	13.83	***	1.19	1.58
Constructive coping		0.56	0.13	.17	4.49	***	0.31	0.81

The all samples composed

*** *p*<.001, ** *p*<.01.

Again, a multiple group analysis was conducted to examine gender differences in the relationship between reassessing coping and depressive symptoms (see Table 3). For genders, the *R* ^2^ values were significant at the *p*<.001 level (women: *R* ^2^  = .17, *F* (3,748) = 51.52, Cohen’s *f* ^2^  = .21; men: *R* ^2^  = .24, *F* (3,744) = 77.13, Cohen’s *f* ^2^  = .31). The beta weights for reassessing coping scores in genders were significant and negative (women: *β* = −.23, *p*<.001; men: *β* = −.35, *p*<.001). There were no significant gender differences in the beta-weight at the *p*<.05 level (*z* = 1.61, *p*  = .054).

Meanwhile, the beta weights for distancing coping scores in all country samples were significant and positive (the United States: *β*  = .41, *p*<.001; Australia: *β*  = .39, *p*<.001; China: *β*  = .46, *p*<.001). The beta-weight difference in distancing coping between the Australian and Chinese samples was significant (*z* = 2.05, *p*<.05), but the differences between the United States and Australian (*z* = 0.55, *p*  = .29) and the United States and Chinese (*z* = 1.49, *p*  = .068) samples were not. Moreover, the beta weights for distancing coping scores in both women and men were significant and positive (women: *β*  = .39, *p*<.001; men: *β*  = .45, *p*<.001). No significant gender difference in the beta-weight was found (*z* = 0.84, *p*  = .20).

The beta weights for constructive coping scores in both the United States and Australian samples were significant and positive (United States: *β*  = .13, *p*<.01; Australia: *β*  = .15, *p*<.001; China: *β*  = .04, *p*  = .43); however, these beta values were small. The beta-weight difference in constructive coping between the Australian and Chinese samples was significant (*z* = 1.91, *p*<.05), but the differences between the United States and Australian (*z* = 0.44, *p*  = .33) and the United States and Chinese (*z* = 1.48, *p*  = .07) samples were not. In addition, the beta weight for constructive coping scores was significant and positive in men (*β*  = .17, *p*<.001), but not in women (*β*  = .07, *p*  = .08). No significant gender difference in the beta-weight was shown (*z* = 1.62, *p*  = .053).

## Discussion

The hypothesis that reassessing coping with interpersonal stressors is associated with lower levels of depressive symptoms was tested for each sample in the United States, Australia, and China via a cross-sectional Web-based survey. The SEM analysis revealed that significant proportions of the variance in depressive symptom scores were accounted for by participants’ scores on all three coping strategies for interpersonal stress in every sample. The effect sizes were medium or large; according to Cohen [38], small, medium, and large effect sizes correspond to *f* ^2^ values of.02,.15, and.35, respectively. In all samples, the beta values for reassessing coping were significant and negative, indicating that our hypothesis was supported. Until now, the effects of reassessing coping on psychological distress have been demonstrated only with specific samples in Japan [8], [10], [11]. However, our result showed that reassessing coping is associated with reduced depressive symptoms also in three populations with different sociocultural values (e.g., collectivistic vs. individualistic). These findings have potential implications for many individuals dealing with interpersonal stressors―the most frequently encountered stressors in daily life that strongly influence levels of depressive symptoms [39]. Consequently, when learning how to cope with interpersonal stressors, reassessing coping may be crucial to reduce their negative effects on depressive symptoms.

On the other hand, distancing coping was significantly associated with a higher level of depressive symptoms in each country. This result was consistent with previous studies in Japanese samples [8], [10], [11] and our hypothesis. In addition, the beta values for this strategy were relatively high (*β*  = .41,.39, and.46) in all of the countries we surveyed. Distancing coping may be a maladaptive strategy in a number of countries. If so, stress management that reduces the use of distancing coping may help in attenuating psychological distress, including depressive symptoms.

Constructive coping was significantly associated with a higher level of depressive symptoms in the United States and Australian samples, but no significant association was found in the Chinese sample. Although our beta values in the United States and Australian samples were small, our findings in these samples were consisted with previous studies in Western cultures [24] that examined relationships between coping strategies similar to constructive coping and psychological distress. The result in the Chinese sample was consistent with those in Japanese samples [8], [10], [11], in which positive but non –significant correlations between constructive coping and depressive symptoms. The effects of constructive coping on depressive symptoms as well as the frequency of use may differ between cultures. However, previous research as well as the current study have not presented the rationale and its evidence for the effects of constructive coping on depressive symptoms or psychological distress; future research should uncover the mechanisms for the effects of constructive coping before any cultural and gender differences are discussed.

Our findings on cross-cultural differences in reassessing and constructive coping indicated that individuals in China scored highest on this approach toward coping with interpersonal stressors, followed by those in the United States and in Australia. These results were consistent with our hypothesis. Therefore, reassessing and constructive coping may be associated with characteristics of collectivistic cultures. These cross-cultural differences in coping strategies for interpersonal stress may be elaborated through further research on understanding cultural differences and counseling research on interpersonal relationships.

Our hypothesis on gender difference in constructive and distancing coping, that women would employ constructive coping more frequently than men, was supported. Others [4], [30] have interpreted gender differences in depression and depressive symptoms by differences in how women and men those in respond to interpersonal stressors. Our findings and future research may contribute to advance research on gender differences in depression.

Several limitations to the present study need to be mentioned. Our findings, obtained through a cross-sectional Web-based survey, need to be interpreted with some caution because this method may produce issues associated with Web-administered surveys as well as limitations with regard to conclusions. Several researchers have asserted a number of methodological issues related to Internet studies [for a review, 40]. However, Gosling et al. [40] suggested that many of these were preconceptions, after comparing a large Internet sample (*N* = 361,703) with a set of 510 published, traditional samples. For example, Internet samples were more diverse than traditional samples with respect to socioeconomic status and geographic location. Furthermore, there was little support for the belief that Internet users were unusually maladjusted; instead, there was evidence that Internet-based findings were not adversely affected by non-serious respondents and were consistent with those drawn from traditional methods of research. Therefore, our data collected through a Web-based survey were useful in understanding reassessing coping with interpersonal stress, although some caution may need to be exercised in the interpretation of the present findings.

Next, our data were gathered through self-reported measures via a cross-sectional design, although a previous study on reassessing coping by Kato [8] employed a longitudinal approach. Causality between coping strategies and depressive symptoms cannot be established from our findings on their associations. According to the transactional theory proposed by Lazarus and colleagues [6], [7], coping behavior affects psychological functioning, including depressive symptoms. This theory’s validity and utility have been supported by numerous studies [6]. Therefore, in the current study, coping strategies might be causal factors, and depressive symptoms, their probable outcomes. Nevertheless, attention should be directed toward interpretations of a causal relationship between coping behavior and depressive symptoms, even with longitudinal data obtained through further research on the topic.

In the current study, we did not define or measure the type of interpersonal relationship (e.g., significant other, acquaintance, and professional relationship) where participants encountered interpersonal stressors. The effects of reassessing coping on depressive symptoms may depend on the degree of closeness between people in a relationship. These issues should be taken into consideration when interpreting our findings.

Despite these limitations, our hypothesis that reassessing coping is negatively associated with depressive symptoms was supported by a Web-based survey of representative in the United States, Australia, and China. We also observed that distancing coping was associated with a high level of depressive symptoms in all countries. In addition to these findings, our data indicated the existence of cross-cultural differences in coping strategies for interpersonal stress. The findings of our study may enable individuals to better deal with distressing interpersonal relationships that they may be experiencing in their daily lives.

## Supporting Information

Dataset S1 **Additional data.**
(XLSX)
